# Sex-Associated Differential mRNA Expression of Cytokines and Its Regulation by Sex Steroids in Different Brain Regions in a *Plasmodium berghei* ANKA Model of Cerebral Malaria

**DOI:** 10.1155/2018/5258797

**Published:** 2018-11-01

**Authors:** Martha Legorreta-Herrera, Karen E. Nava-Castro, Margarita I. Palacios-Arreola, Rosalía Hernández-Cervantes, Jesús Aguilar-Castro, Luis A. Cervantes-Candelas, Jorge Morales-Montor

**Affiliations:** ^1^Laboratorio de Inmunología Molecular, Facultad de Estudios Superiores Zaragoza, Universidad Nacional Autónoma de México, Ciudad de México 09230, Mexico; ^2^Laboratorio de Genotoxicología y Medicina Ambientales, Departamento de Ciencias Ambientales, Centro de Ciencias de la Atmósfera, Universidad Nacional Autónoma de México, Ciudad de México 04510, Mexico; ^3^Departamento de Inmunología, Instituto de Investigaciones Biomédicas, AP 70228, Universidad Nacional Autónoma de México, Ciudad de México 04510, Mexico

## Abstract

Cerebral malaria (CM) is the major complication associated with death in malaria patients, and its pathogenesis is associated with excessive proinflammatory cytokine production. Notably, the severity and mortality of natural infections with *Plasmodium* are higher in males than females, suggesting that sexual hormones influence both the pathogenesis of and immune response in CM. However, no studies on inflammation mediators in the brains of both sexes have been reported. In this work, the mRNA expression levels of the proinflammatory cytokines IL-1*β*, IFN-*γ*, TNF-*α*, and IL-2 were measured in the preoptic area, hypothalamus, hippocampus, olfactory bulb, frontal cortex, and lateral cortex regions of gonadectomized female and male CBA/Ca mice infected with *P. berghei* ANKA (a recognized experimental CM model). Our findings demonstrate that both infection with *P. berghei* ANKA and gonadectomy trigger a cerebral sex dimorphic mRNA expression pattern of the cytokines IL-1*β*, TNF-*α*, IFN-*γ*, and IL-2. This dimorphic cytokine pattern was different in each brain region analysed. In most cases, infected males exhibited higher mRNA expression levels than females, suggesting that sexual hormones differentially regulate the mRNA expression of proinflammatory cytokines in the brain and the potential use of gonadal steroids or their derivates in the immunomodulation of cerebral malaria.

## 1. Introduction

Malaria is one of the most important health problems in the world; in 2016, malaria killed 445,000 people, mainly targeting children under 5 years old [1]. The major malaria complication is cerebral malaria (CM), a neurological syndrome that mainly affects children under 5 years old and immunocompromised individuals [2]. Two hypotheses exist to explain the pathogenesis of CM: mechanical obstruction and immunopathology. Mechanical obstruction involves the microvascular obstruction of parasitised red blood cells [3] and is associated with microvascular thrombosis [4], loss of endothelial barrier function [5] and endothelial dysregulation [6]. The immunopathology hypothesis is characterized by excessive proinflammatory cytokine production [7, 8] and suggests that CM results from an exacerbated immune response originally developed for host protection, wherein proinflammatory cytokines and immune system effector cells have a central role. Consistent with this theory, *in vivo* removal of CD4^+^ T cells by monoclonal antibodies [9] and neutralization of the proinflammatory cytokines IFN-*γ*, TNF-*α*, and IL-1*β* are directly associated with protection against CM development [10–12].

Notably, the severity and mortality of natural infections with *Plasmodium* are higher in males than females [13–15]. In experimental malaria models, gonadectomy and hormone replacement therapy show that the susceptibility of male mice is determined by gonadal steroids [16, 17]. Furthermore, castration of male mice induces resistance to *Plasmodium* infection, while the administration of testosterone or oestradiol induces immunosuppression [16, 18, 19]. In addition, male sex hormones modulate the function of Th1/Th2 cytokines in the spleen, driving differences in susceptibility to *Plasmodium* infection [20]. These studies show that the levels of sexual hormones influence both the course of *Plasmodium* infection and the immunomodulation of malaria pathogenesis. However, whether sexual hormones are involved in the pathogenesis of CM remains unclear.

CBA/Ca mice infected with *Plasmodium berghei* ANKA have many characteristics in common with human diseases and serve as the best CM model available. As in humans, parasitised red blood cells accumulate in the brains of susceptible mice during infection, and numerous leukocytes are present in the brain blood vessels of these mice [21, 22].

In previous studies, we have shown sexual dimorphism in the systemic immune responses of CBA/Ca mice infected with *P. berghei* ANKA [17]. Sex steroids are important modulators of the immune system; oestrogen, progesterone, and testosterone regulate different immune cell functions, such as growth, differentiation, and survival. The presence of sex steroid receptors on immune cells indicates that interactions with their ligands are important for their function [23]. In addition, CM is characterized by the increased systemic production of proinflammatory cytokines, such as TNF-*α*, IFN-*γ*, and IL-1*β* [9, 24], and previous studies suggest that nitric oxide (NO) may also contribute to the pathogenesis of CM [25]. Furthermore, an inverse correlation has been observed between the levels of NO in plasma and the incidence of severe malaria in human populations [26, 27]. However, despite knowledge gained during several decades of CM research using both postmortem analysis and experimental models, the mechanisms by which hormones modulate sexual dimorphism in CM are unknown. In this work, we analysed the effects of sex and gonadectomy on the mRNA expression of proinflammatory cytokines in specific brain areas of mice infected with *P. berghei* ANKA.

To our knowledge, this work is the first report documenting the dimorphic and sex steroid-regulated expression of proinflammatory cytokines in delimited areas of the brain during CM. The dimorphic mRNA expression of IL-1*β*, IL-2, TNF-*α*, and IFN-*γ* in the brain, may at least partially explain sexual dimorphism in CM.

## 2. Materials and Methods

### 2.1. Mice

CBA/Ca mice were originally donated by Dr. William Jarra (National Institute for Medical Research, London, UK). Animal care and experiments were conducted at the FES Zaragoza, Universidad Nacional Autónoma de México (UNAM). All experimental procedures in the animals were approved by the Institutional Care and Animal Use Committee, permit number 28/04/SO/3.4.1, and adhered to Mexican regulation NOM-062-ZOO-1999 for the use and care of laboratory animals. Euthanasia of the experimental animals was performed humanely by cervical dislocation after anaesthesia with 5% sevoflurane (Abbot, México).

### 2.2. Parasites and Infection

*P. berghei* ANKA parasites were also kindly donated by Dr. Jarra and were cryopreserved in liquid nitrogen. The parasites were thawed and injected into one 4-week-old mouse. When parasitaemia reached 20%, parasitised red blood cells were used to infect the experimental CBA/Ca mice. All the infected animals were intravenously inoculated with 1 × 10^3^*P. berghei* ANKA-infected red blood cells.

### 2.3. Ovariectomy

Ovariectomies were performed as we previously reported [17]. Briefly, four-week-old female mice were anaesthetised with ketamine (80 mg/kg body weight, Phoenix Pharmaceutical Inc., St. Joseph, MO, US), and incisions were made in the lower abdomen. The ovaries were removed, and the abdomen was sutured. Sham-operated animals underwent an identical procedure without ovary removal. The mice were allowed to recover from surgery for 4 weeks and were then infected with *P. berghei* ANKA. Mice were sacrificed 9 days after infection; the absence of ovaries was confirmed by visual inspection. The complete design was repeated two times using 5 female mice per treatment in each individual experiment (*n* = 10).

### 2.4. Orchiectomy

Four-week-old male mice were anaesthetised, and their testes were removed via scrotal incisions as we previously reported [17]. Briefly, the ductuli efferent was transected by electrocauterisation, and the testes and epididymis were removed. Sham-operated mice underwent an identical procedure without removal of the testes. The complete design was repeated two times using 5 male mice per treatment in each individual experiment (*n* = 10).

Mice were allocated into three groups (*n* = 10) for each sex: (1) intact mice (*n* = 10), (2) bilaterally gonadectomized (GX) (*n* = 10) mice, and (3) mice that underwent surgery without gonadectomy (sham operated) (*n* = 10). The mice were allowed to recover from surgery for 4 weeks prior to parasite infection. The mice were sacrificed by cervical dislocation on day 9 postinfection, and their blood and brains were collected to assess parasitaemia and proinflammatory cytokine mRNA expression, respectively.

### 2.5. Parasitaemia

On days 5 to 9 postinfection, thin blood smears were prepared, fixed with absolute methanol, and stained with a 1 : 10 dilution of Giemsa stain (Sigma-Aldrich, St. Louis, MO, US) in Giemsa buffer. Enumeration of the parasite load was performed under a 100x oil immersion lens using a Zeiss Standard 20 microscope (Carl Zeiss Ltd., Welwyn Garden City, UK). Parasitaemia levels of 0.5% or greater were evaluated by counting the number of parasitised erythrocytes that were present among 200 red blood cells. Lower levels of parasitaemia were determined by counting the number of parasitised red blood cells that were present in 50 microscope fields. The data are presented as the geometric mean of the percentage of parasitaemia. The complete experiment was repeated 2 times (10 animals per group).

### 2.6. Collection and Processing of Brain Tissues

The preoptic area (POA), hypothalamus (HT), olfactory bulb (OB), hippocampus (HC), frontal cortex (FC), and lateral cortex (LC) regions from all mice were extracted according to *The Mouse Brain in Stereotaxic Coordinates*. Briefly, the POA and HT regions were dissected by making a razor cut to the anterior commissure just rostral to the optic chiasm, the anteroventral limit of which was the nucleus of the diagonal band of the lateral Broca. The HC was obtained by making a razor cut underneath the FC, and the FC was excised directly from the frontal lobes; the LC was excised directly from the lateral lobes.

Brain tissues from 5 animals were pooled by region to increase the sample quantity. All experiments were performed in duplicate (*n* = 10).

### 2.7. RNA Extraction

Total RNA was isolated from the POA, HT, HC, OB, FC, and LC sections of control and infected mice using TRIzol (Gibco-BRL, NY, USA). Briefly, each tissue sample was removed and disrupted immediately in TRIzol (1 mL/0.1 g tissue); 0.2 mL of chloroform was then added per 1 mL of TRIzol. The aqueous phase was recovered after 10 minutes of centrifugation at 14,000 ×g. RNA was precipitated with isopropyl alcohol, washed with 75% ethanol, and dissolved in RNase-free water. RNA concentrations were measured, based on absorbance at 260 nm, and RNA purity was verified after electrophoresis on 1.0% denaturing agarose gel in the presence of 2.2 M formaldehyde. Total RNA from all extracted tissues was reverse transcribed, and IL-1*β*, IL-2, IFN-*γ*, TNF-*α*, and 18S ribosomal RNA (control) were then PCR-amplified from the resulting cDNAs.

### 2.8. IL-1*β*, IL-2, IFN-*γ*, and TNF-*α* mRNA Expression in Brain Tissues

The sequences of primers used for amplification have been previously published [28]. Briefly, 1 *μ*g of total RNA from each tissue was incubated for 1 h at 37°C with 400 units of M-MLV reverse transcriptase (Applied Biosystems, Boston MA, USA) in a 50 *μ*L reaction, containing 50 mM of each dNTP and 0.05 *μ*g of oligo (dT) primer (Gibco). The 50 *μ*L PCR reaction contained 10 *μ*L of cDNA, 5 *μ*L of 10x PCR buffer (Biotecnologias Universitarias, México), 1 mM of MgCl_2_, 0.2 mM of each dNTP, 0.05 *μ*M of each primer, and 2.5 units of Taq DNA polymerase (Biotecnologías Universitarias).

Twenty microliters of each PCR reaction was electrophoresed on a 2% agarose gel and visualized with ethidium bromide. A single band was detected in all cases, as expected. To determine whether all reactions were in the exponential phase of amplification and ensure that the changes in expression were not artefacts (e.g., 18S rRNA in the stationary phase), we constructed RNA, cycling, and temperature curves for each gene.

### 2.9. Densitometric Analysis

PCR bands were quantified by the densitometric scanning of several autoradiograms at various exposures and expressed as the ratio of the target gene (cytokines) signal to that of 18S rRNA.

### 2.10. Nitric Oxide Measurement

Serum nitrate concentrations were evaluated according to the Griess method as described previously [29]. Briefly, 15 *μ*L of serum from each mouse was incubated with 5 *μ*L of nitrate reductase (5 U/mL; Boehringer Mannheim, Laval, Quebec, Canada) and 15 *μ*L of NADPH (1.25 mg/mL; Sigma-Aldrich) for 15 min at room temperature; 100 *μ*L of Griess reagent (Sigma-Aldrich) and 100 *μ*L of trichloroacetic acid (10% aqueous solution) were added. The protein precipitate was then removed by centrifugation at 14,000 rpm for 5 min, and 100 *μ*L of each supernatant was transferred to a 96-well flat-bottom plate. Concentrations were evaluated by an ELISA reader (Stat Fax Plate Translator, NY, USA) at 540 nm using a standard curve comprising sodium nitrate (Sigma-Aldrich) diluted in pooled serum from uninfected control mice treated in the same manner as the experimental samples.

### 2.11. Experimental Design and Statistical Analysis

We designed the study as a 3-factor experiment. The independent variables were infection (yes, no), sex (male or female), and gonadectomy (yes, no). The dependent variables were the number of parasites and the mRNA expression levels of IL-1*β*, IL-2, IFN-*γ*, and TNF-*α* in each tissue sample, expressed as the ratio of the optical density (OD) of the corresponding gel to that of 18S rRNA. Additionally, we measured nitric oxide levels in plasma.

The complete design was repeated 2 times using 20 mice per group (GX, sham-operated, or intact female and male mice). Half of the mice in each group (*n* = 10) were infected with *P. berghei* ANKA, and the other half served as uninfected controls. Statistical analysis of variance was performed using Prism 7 software (GraphPad Software Inc.). All data are expressed as the mean ± SD of two independent experiments. When applied, post hoc individual contrasts of group means were analysed by Tukey's test using the sum of residual and 3-factor interaction variances to test for significant differences. A *P* value < 0.05 indicated a significant difference.

## 3. Results

### 3.1. Gonadectomy Increased Parasitaemia in CBA/Ca Male Mice Infected with *P. berghei* ANKA

**Figure 1:**
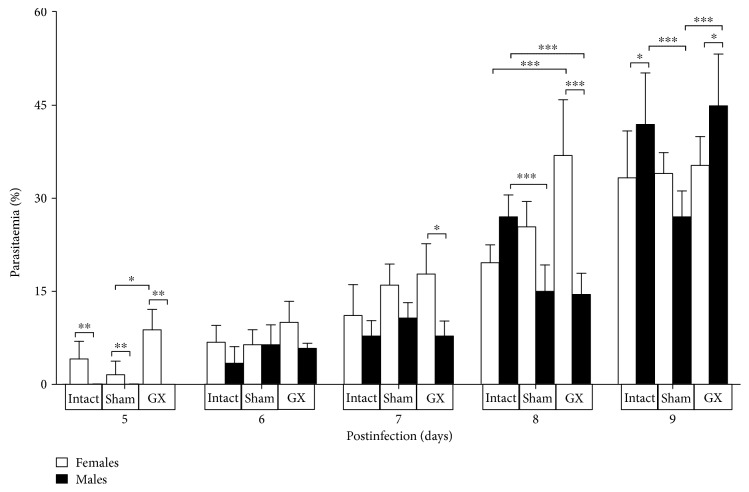
Effect of gonadectomy on parasitaemia in CBA/Ca mice infected with *P. berghei* ANKA. Intact, sham operated, or gonadectomized (GX) female and male mice were infected with *P. berghei* ANKA. Parasitaemia was measured using Giemsa-stained blood films from day 5 to 9 postinfection. Values are presented as the geometric means ± SD (*n* = 10). The data are representative of two independent experiments, with five mice in each experiment (*n* = 10) per group. ^∗^*P* ≤ 0.01, ^∗∗^*P* ≤ 0.05, and ^∗∗∗^*P* ≤ 0.001. ANOVA and Tukey's test were performed to compare the selected pairs of columns.

Parasitaemia in intact female mice was higher than in intact male mice on days 5, 6, and 7 postinfection. However, this dimorphic pattern changed on day 9 postinfection, at which time intact female mice showed lower levels of parasitaemia than their male counterparts. Parasitaemia was significantly higher in both intact and GX male mice compared with that in their female counterparts on day 9 postinfection. Interestingly, in sham-operated mice, parasitaemia was significantly higher in female mice than in males (Figure 1).

### 3.2. Effects of Sex and Gonadectomy on Cytokine mRNA Expression in the Preoptic Area (POA) of *P. berghei* ANKA-Infected Mice

**Figure 2:**
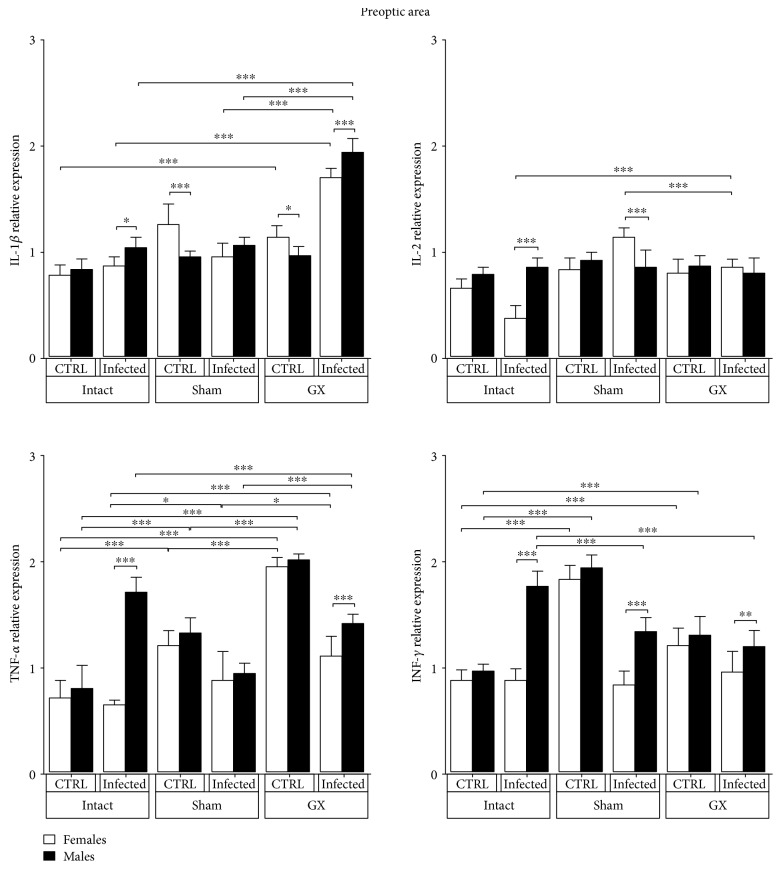
Preoptic area (POA) expression of IL-1*β*, IL-2, TNF-*α*, and IFN-*γ* in CBA/Ca male and female mice in control (CTRL) and *Plasmodium berghei* ANKA-infected groups in response to manipulation (sham group) or surgery (gonadectomy). Gonadectomized (GX) or intact male and female mice were infected with *P. berghei* ANKA and sacrificed on day 9 postinfection. POAs were dissected from their brains, and RNA was isolated and reverse transcribed. Complementary DNA (cDNA) was used to PCR amplify IL-1*β*, IL-2, TNF-*α*, and IFN-*γ* related to 16S RNA. Data are presented as the means ± SD. ^∗^*P* < 0.05, ^∗∗^*P* < 0.01, and ^∗∗∗^*P* < 0.001. ANOVA and Tukey's test were performed to compare the selected pairs of columns. The data are representative of two independent experiments using five mice in each experiment, with *n* = 10 mice per group.

We measured the relative mRNA expression levels of the proinflammatory cytokines IL-1*β*, TNF-*α*, and IFN-*γ* and the Th1 cytokine IL-2 in the POA of intact, sham, or GX female and male mice, either uninfected or infected with *P. berghei* ANKA. All cytokines were detected in control and infected mice of both sexes in the brain areas analysed, and the levels varied by the gonadectomy and infection status. Gonadectomy significantly upregulated the mRNA expression of IL-1*β* in the infected animals of both sexes compared to that in their respective intact groups. Interestingly, infected males had a higher increase in IL-1*β* expression (0.5-fold, *P* < 0.05) than their female counterparts in the same condition (Figure 2).

Infection clearly induced a dimorphic pattern; it reduced IL-2 mRNA expression in intact females compared to that in intact males while decreasing IL-2 mRNA expression in sham-infected males compared to that in sham-infected females (*P* < 0.05) (Figure 2). Infection also induced a 3.0-fold increase in TNF-*α* mRNA expression in intact males compared to that in intact females (*P* < 0.01). Interestingly, gonadectomy increased TNF-*α* mRNA expression in the uninfected mice of both sexes (*P* < 0.01), and infection in GX mice significantly downregulated its expression (*P* > 0.01) (Figure 2). Finally, infection also induced a dimorphic pattern in IFN-*γ* mRNA expression, with significantly upregulated expression in intact, sham-, and GX-infected males compared with that in their female counterparts in the same condition (*P* < 0.01) (Figure 2).

### 3.3. Effects of Sex and Gonadectomy on Cytokine mRNA Expression in the Olfactory Bulbs of CBA/Ca Mice Infected with *P. berghei* ANKA

**Figure 3:**
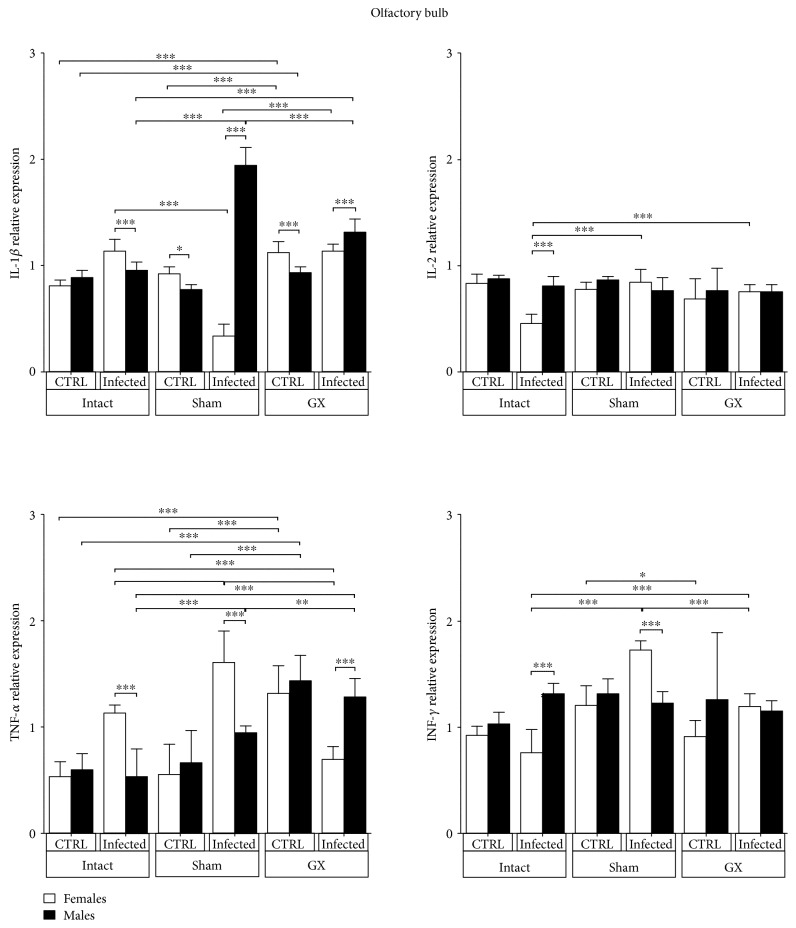
Olfactory bulb (OB) expression of IL-1*β*, IL-2, TNF-*α*, and IFN-*γ* in CBA/Ca male and female mice in control (CTRL) and *Plasmodium berghei* ANKA-infected groups in response to manipulation (sham group) or surgery (gonadectomy). Gonadectomized (GX) or intact male and female mice were infected with *P. berghei* ANKA and sacrificed 9 days postinfection. OBs were dissected from their brains, and RNA was isolated and reverse transcribed. Complementary DNA (cDNA) was used to amplify IL-1*β*, IL-2, TNF-*α*, and IFN-*γ* relative to 18S RNA via PCR. Data are presented as the means ± S.D. ^∗^*P* < 0.05, ^∗∗^*P* < 0.01, and ^∗∗∗^*P* < 0.001. ANOVA and Tukey's test were performed to compare the selected pairs of columns. The data are representative of two independent experiments using five mice in each experiment, with *n* = 10 mice per group.

IL-1*β*, IL-2, TNF-*α*, and IFN-*γ* were detected in the OBs of mice of both genders and in control intact and infected mice. IL-1*β* mRNA expression was constant in intact female and male mice. Infection induced a dimorphic pattern, particularly in sham-infected animals; females exhibited significantly decreased IL-1*β* expression compared with that in sham, intact, and uninfected mice, and the opposite effect was detected in males (*P* < 0.001) (Figure 3). IL-2 mRNA expression was decreased in intact infected females compared with that in intact infected males, inducing a dimorphic pattern in only these groups (*P* < 0.05) (Figure 3). Infection significantly increased TNF-*α* expression in intact and sham females compared with their counterpart males in the same condition, inducing a sex-associated pattern. TNF-*α* mRNA expression was increased 3.0- and 1-fold in sham-infected females and males, respectively, compared with their respective sham uninfected controls. In contrast, in the GX-infected groups, dimorphic TNF-*α* expression was detected; females exhibited significantly decreased TNF-*α* mRNA expression, while TNF-*α* expression was not altered in males compared to that in their GX uninfected counterparts (*P* < 0.05) (Figure 3). Finally, infection induced a dimorphic IFN-*γ* mRNA expression pattern in only intact males; these mice exhibited upregulated expression of this gene compared to that in intact infected female mice (*P* < 0.05) (Figure 3).

### 3.4. Effects of Sex and Gonadectomy on IL-1*β*, IL-2, TNF-*α*, and IFN-*γ* mRNA Expression in the Hypothalamus of CBA/Ca Mice Infected with *P. berghei* ANKA

**Figure 4:**
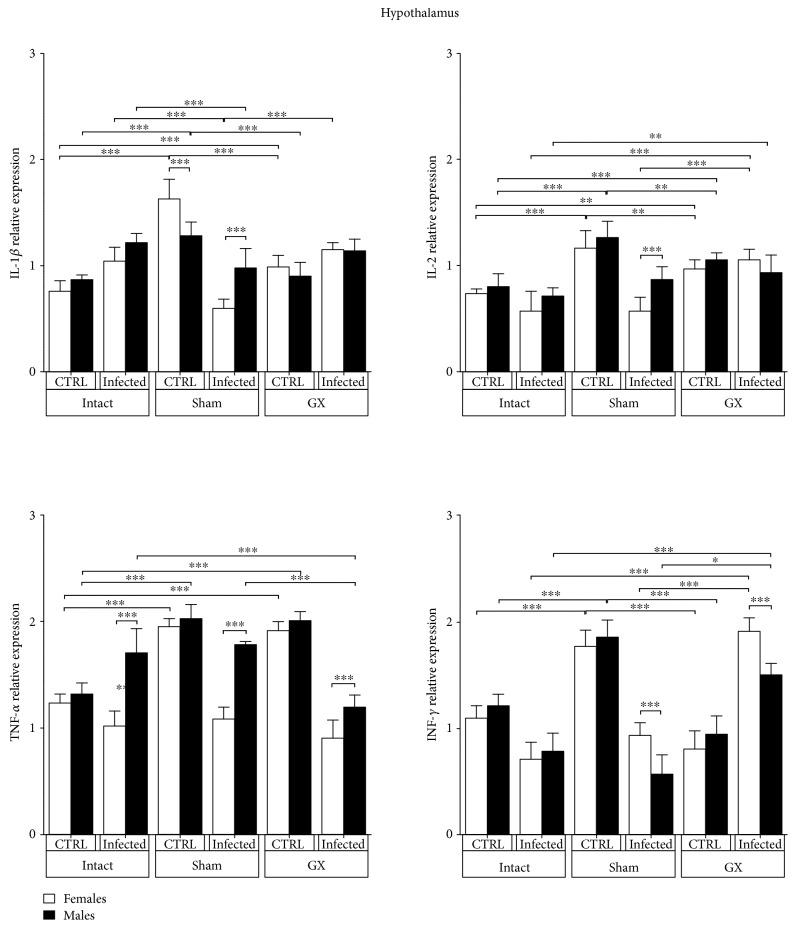
Hypothalamus (HT) expression of IL-1*β*, IL-2, TNF-*α*, and IFN-*γ* in CBA/Ca male and female mice, control (CTRL), and *Plasmodium berghei* ANKA-infected groups in response to manipulation (sham group) or surgery (gonadectomy). Gonadectomized (GX) or intact male and female mice were infected with *P. berghei* ANKA and sacrificed 9 days postinfection. The HT regions were dissected from their brains, and RNA was isolated and reverse transcribed. Complementary (cDNA) was used to PCR-amplify IL-1*β*, IL-2, TNF-*α*, and IFN-*γ* relative to 18S RNA. The data are presented as the means ± SD. ^∗^*P* < 0.05, ^∗∗^*P* < 0.01, and ^∗∗∗^*P* < 0.001. ANOVA and Tukey's test were performed to compare the selected pairs of columns. The data are representative of two independent experiments using five mice in each experiment, with *n* = 10 mice per group.

No differences in IL-1*β* expression were detected between uninfected intact mice of either gender, a pattern that was not altered by infection or gonadectomy. However, in the sham groups, IL-1*β* expression was altered and became sex related; sham uninfected mice exhibited increased IL-1*β* expression, and the expression of this gene was higher in females compared to that in their male counterparts. *P. berghei* ANKA infection decreased IL-1*β* expression in sham females and did not alter its expression in males (*P* < 0.05) (Figure 4). IL-2 mRNA expression was dimorphic only in sham-infected animals and was not affected by infection (Figure 4). TNF-*α* mRNA expression in the HT was detectable in all groups. Interestingly, infection upregulated TNF-*α* expression in the males of the intact, sham, and GX groups, generating a dimorphic pattern (*P* < 0.001) (Figure 4). IFN-*γ* expression was not sex associated. However, sham and gonadectomy surgeries in infected animals induced a dimorphic IFN-*γ* expression pattern, with significantly higher expression in females than in males (*P* < 0.001) (Figure 4).

### 3.5. Effects of Sex and Gonadectomy on Cytokine mRNA Expression in the Hippocampus of CBA/Ca Mice Infected with *P. berghei* ANKA

**Figure 5:**
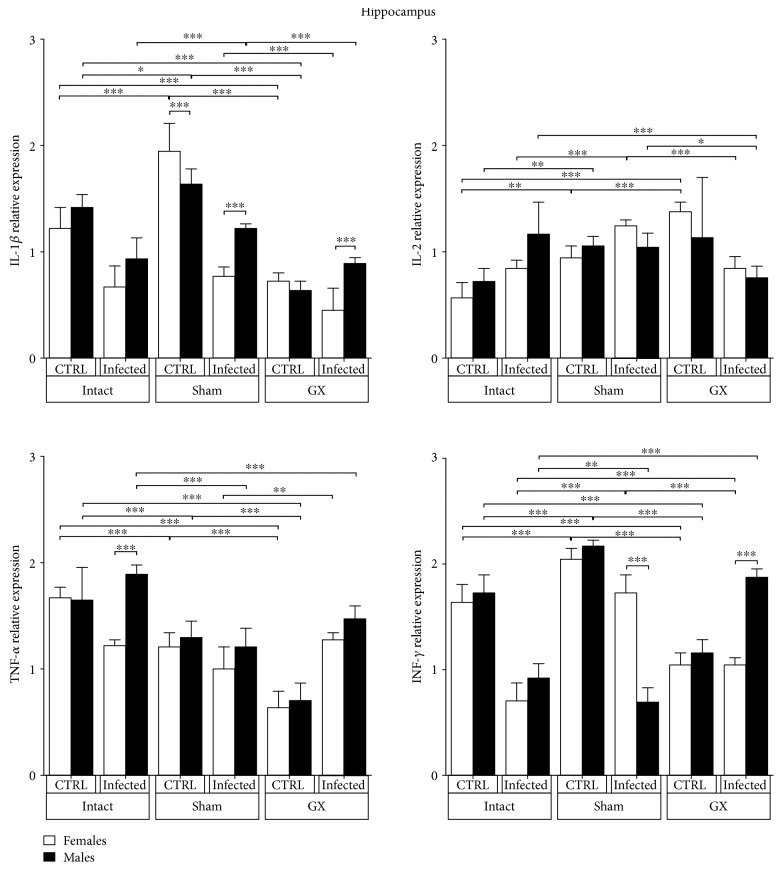
Sex-associated expression of IL-1*β*, IL-2, TNF-*α*, and IFN-*γ* in the hippocampus (HC) of CBA/Ca male and female mice in the control (CTRL) and *Plasmodium berghei* ANKA-infected groups in response to manipulation (sham groups) or surgery (gonadectomy). Gonadectomized (GX) or intact male and female mice were infected with *P. berghei* ANKA and sacrificed 9 days postinfection. The HC regions were dissected from their brains, and RNA was isolated and reverse transcribed. Complementary DNA (cDNA) was used to PCR amplify IL-1*β*, IL-2, TNF-*α*, and IFN-*γ* relative to 18S RNA. The data are presented as the mean ± SD. ^∗^*P* < 0.05, ^∗∗^*P* < 0.01, and ^∗∗∗^*P* < 0.001. ANOVA and Tukey's test were performed to compare the selected pairs of columns. The data are representative of two independent experiments using five mice in each experiment, with *n* = 10 mice per group.

IL-1*β* mRNA expression did not differ between sexes and did not change with infection. However, in the sham-infected groups, a clear sexual dimorphism was detected; IL-1*β* expression was higher in infected males than in infected females (*P* < 0.001) (Figure 5). IL-2 mRNA expression remained steady in all the groups analysed (Figure 5). TNF-*α* mRNA expression was equally expressed in males and females in all groups except the intact groups. In these groups, sex-associated dimorphism was detected depending on the infection status; TNF-*α* expression was decreased in females and increased in males (*P* < 0.001) (Figure 5). IFN-*γ* expression was similar between genders in the intact, sham, and GX uninfected mice. Interestingly, infection decreased IFN-*γ* mRNA expression in intact mice and induced a sex-dependent pattern in the sham and GX groups. IFN-*γ* mRNA expression was higher in sham-infected female mice than in males in the same condition. In the GX-infected groups, females exhibited significantly lower IFN-*γ* expression compared to that in their male counterparts in the same condition (*P* < 0.001) (Figure 5).

### 3.6. Effects of Sex and Gonadectomy on Cytokine mRNA Expression in the Lateral Cortex of CBA/Ca Mice Infected with *P. berghei* ANKA

**Figure 6:**
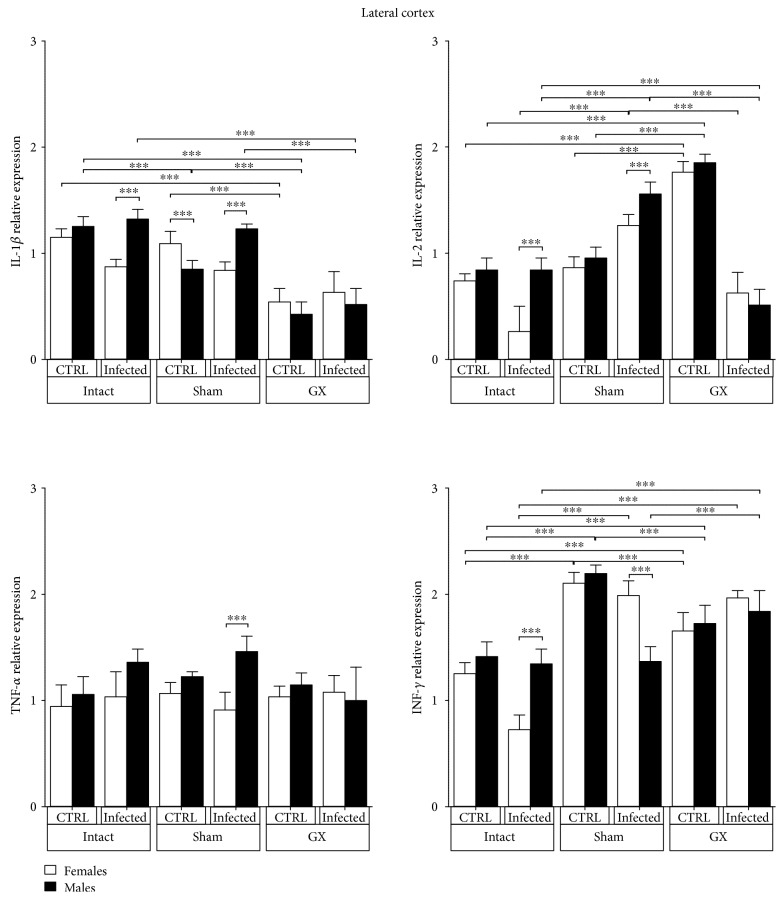
Lateral cortex (LC) expression of IL-1*β*, IL-2, TNF-*α*, and IFN-*γ* in CBA/Ca male and female mice in control (CTRL) and *Plasmodium berghei* ANKA-infected groups in response to manipulation (sham groups) or surgery (gonadectomy). Gonadectomized (GX) or intact male and female mice were infected with *P. berghei* ANKA and sacrificed 9 days postinfection. The LC regions were dissected from their brains, and RNA was isolated and reverse transcribed. Complementary DNA (cDNA) was used to PCR amplify IL-1*β*, IL-2, TNF-*α*, and IFN-*γ* relative to 18S RNA. The data are presented as the mean ± SD. ^∗^*P* < 0.05, ^∗∗^*P* < 0.01, and ^∗∗∗^*P* < 0.001. ANOVA and Tukey's test were performed to compare the selected pairs of columns. The data are representative of two independent experiments using five mice in each experiment, with *n* = 10 mice per group.

IL-1*β* mRNA expression was dimorphic in intact infected and sham-infected groups; males exhibited higher IL-1*β* mRNA expression than females in the same condition. IL-2 mRNA expression exhibited a dimorphic pattern; it was upregulated in intact infected and sham-infected males compared with their female counterparts. Interestingly, infection downregulated IL-2 mRNA expression in intact females compared with that in males in the same condition and also in GX-infected mice of both sexes (*P* < 0.001) (Figure 6). TNF-*α* expression was similar in all groups, and surgery or infection did not induce gender-related differences except in the sham-infected groups; males exhibited higher TNF-*α* mRNA expression compared to that in females in the same condition (*P* < 0.001) (Figure 6). IFN-*γ* mRNA expression was similar among genders in the intact, sham, and GX uninfected groups. Infection induced a sex-associated pattern; IFN-*γ* mRNA expression was downregulated in intact females and increased in sham female mice (*P* < 0.001) (Figure 6).

### 3.7. Effects of Sex and Gonadectomy on Cytokine mRNA Expression in the Frontal Cortex of CBA/Ca Mice Infected with *P. berghei* ANKA

**Figure 7:**
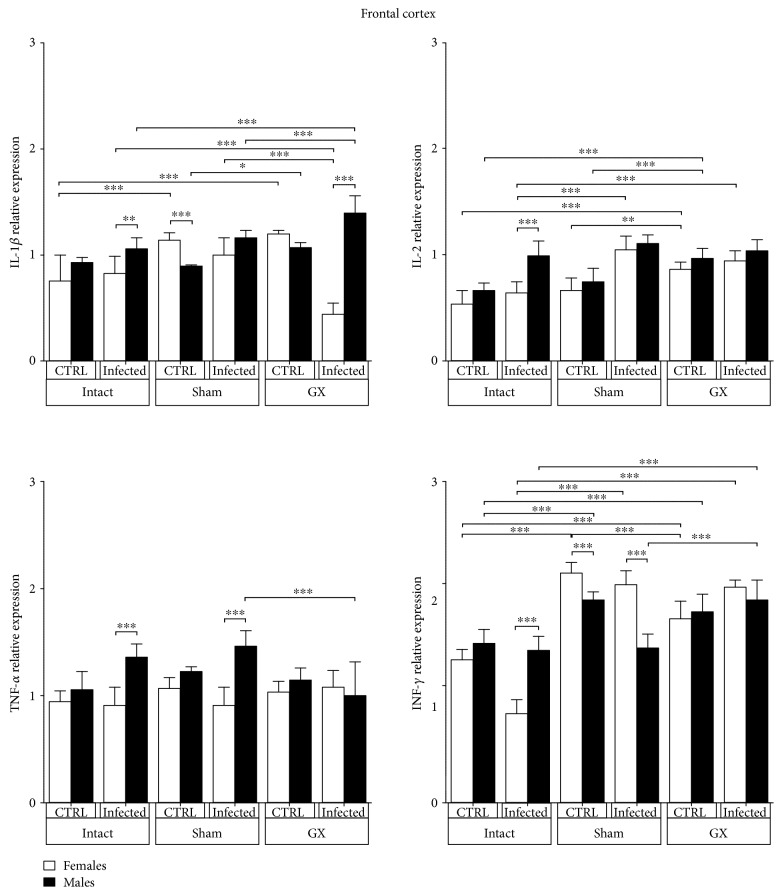
Sex-associated expression of IL-1*β*, IL-2, TNF-*α*, and IFN-*γ* in the frontal cortex (FC) of CBA/Ca male and female mice in control (CTRL) *Plasmodium berghei* ANKA-infected groups in response to manipulation (sham groups) or surgery (gonadectomy). Gonadectomized (GX) or intact male and female mice were infected with *P. berghei* ANKA and sacrificed 9 days postinfection. The FC regions were dissected from their brains, and RNA was isolated and reverse transcribed. Complementary DNA (cDNA) was used to PCR amplify IL-1*β*, IL-2, TNF-*α*, and IFN-*γ* relative to 18S RNA. The data are presented as mean ± SD. ^∗^*P* < 0.05, ^∗∗^*P* < 0.01, and ^∗∗∗^*P* < 0.001. ANOVA and Tukey's test were performed to compare the selected pairs of columns. The data are representative of two independent experiments using five mice in each experiment, with *n* = 10 mice per group.

IL-1*β* was not dimorphic in any group analysed except for GX-infected animals, which clearly showed a dimorphic pattern; IL-1 *β* mRNA expression was significantly higher in males than in females in the same condition (*P* < 0.05) (Figure 7). IL-2 mRNA expression was dimorphic in only intact infected mice, and males exhibited significantly higher expression than females in the same condition (*P* < 0.001) (Figure 7). TNF-*α* mRNA expression was clearly dimorphic in the intact and sham-infected groups, as it was significantly higher in males than in females in the same condition (*P* < 0.001) (Figure 7). IFN-*γ* mRNA expression was dimorphic in the intact and sham-infected groups, and intact infected males exhibited significantly higher IFN-*γ* mRNA levels than intact infected females. In contrast, sham-infected females exhibited higher IFN-*γ* expression than males in the same condition (*P* < 0.001) (Figure 7).

Importantly, in all the experimental groups analysed, IL-4, IL-10, IL-6, and IL-12 were undetectable in every area of the brain analysed. In the positive control (spleens of intact infected mice), these cytokines were always detected (results not shown), suggesting that these findings are not artefacts.

### 3.8. Serum Nitric Oxide Concentration Is Affected by Sex

**Figure 8:**
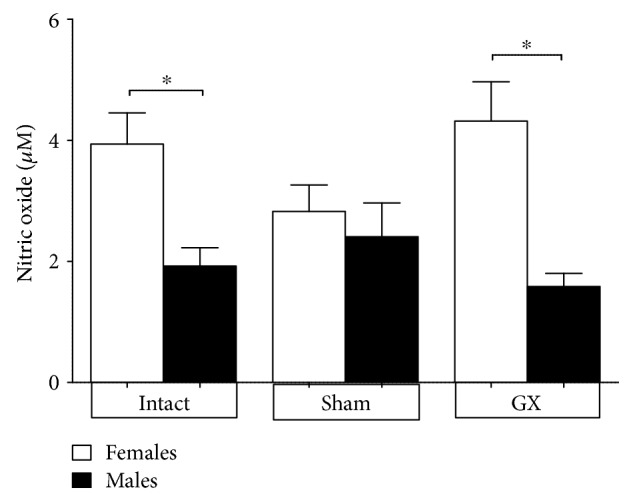
Effect of gonadectomy on serum nitric oxide levels in *Plasmodium berghei* ANKA-infected mice. Gonadectomized (GX) or intact female and male mice were infected with *P. berghei* ANKA and sacrificed 9 days postinfection. Their blood was collected to separate serum, which was used to quantify nitric oxide concentrations using the Griess reaction. The data are presented as the mean ± SD. ^∗^*P* < 0.05. ANOVA and Tukey's test were performed to compare the selected pairs of columns. The data are representative of two independent experiments using five mice in each experiment, with *n* = 10 mice per group.

Nitric oxide is promptly oxidized to the stable inorganic nitrogen oxides nitrite and nitrate *in vivo* [30, 31], and measurement of these metabolites has been used to demonstrate cytokine-inducible nitric oxide synthesis [32]. Nitric oxide levels in this work were significantly higher in both intact females and GX females compared to those in male mice in the same condition (Figure 8).

## 4. Discussion

In this work, we report that both infection with *P. berghei* ANKA and gonadectomy triggered a sexually dimorphic cerebral mRNA expression pattern of the cytokines IL-1*β*, TNF-*α*, IFN-*γ*, and IL-2. This dimorphic cytokine pattern was different in each brain region analysed (POA, HT, HC, OB, FC, and LC). In most cases, infected males exhibited higher mRNA expression levels than females.

Previously, we have documented that infection with *P. berghei* ANKA is lethal to CBA/Ca mice; all female and male infected mice died by day 13 postinfection. Gonadectomy, which substantially decreases sex hormone levels, decreases survival in infected female mice by one day compared with intact female mice. In contrast, gonadectomy in male mice increased survival by one day relative to the intact male group. Interestingly, gonadectomy increased the splenic index only in intact infected male mice, suggesting that gonadal male sex hormones negatively regulate the proliferation of spleen cells in mice infected with *P. berghei* ANKA [17]. These findings can be explained because androgens induce lymphoid atrophy and lymphoid hyperplasia [33]. The spleen is important in malaria because it is the organ where parasitised red blood cells are eliminated. In this work, parasitaemia was higher in intact or gonadectomized female mice on days 5, 6, and 7 postinfection; however, on day 9, male intact or gonadectomized mice exhibited higher parasitaemia than their female counterparts. In addition, gonadectomy increased parasitaemia only in male mice (Figure 1).

Proinflammatory cytokines, such as IFN-*γ*, TNF-*α*, and IL-1*β*, contribute to CM pathogenesis in humans [34] and rodents [35]. In this work, TNF-*α* mRNA expression was upregulated in the POA, HT, LC, FC, and HC of male infected mice compared to that in their female counterparts. Contrary to our expectations, infection downregulated IL-1*β*, TNF-*α*, and IFN-*γ* mRNA expression in some cerebral regions, such as the OB and HC, particularly in the sham and GX mice, suggesting that strong regulatory mechanisms prevent inflammatory immune responses in these specific brain regions. In contrast, gonadectomy upregulated IL-1*β* expression in the POA region, TNF-*α* expression in the POA, HT, OB, and FC regions, and IFN-*γ* expression in the HT region. These findings suggest that gonadal hormones negatively modulate the expression of IL-1*β*, TNF-*α*, and IFN-*γ* in these brain regions. Interestingly, infection triggered dimorphic mRNA expression of proinflammatory cytokines; in general, males exhibited higher expression levels than females. IFN-*γ* was upregulated in the POA, OB, HC, FC, and LC regions of infected male animals compared with that in their female counterparts. These findings are consistent with those of Hunt et al., who reported that IFN-*γ* mRNA is strongly expressed in the cortex of C57Bl/6 mice infected with *P. berghei* ANKA [36]. However, these authors did not compare IFN-*γ* mRNA expression between sexes and performed even less extensive comparisons in GX mice. In our experiments, *P. berghei* ANKA infection in GX mice upregulated IFN-*γ* mRNA expression in the HT and HC, suggesting that gonadal steroids downregulate IFN-*γ* expression in these specific brain regions. In fact, 17*β*-oestradiol, produced primarily by the ovaries in females, is recognized as a neuroprotector in both acute and chronic neurodegenerative disorders, such as cerebral ischaemia, traumatic brain injury, and Alzheimer's disease [37–39]. Our results are also in agreement with those of Linares et al. who found that IFN-*γ* and TNF-*α* mRNA expression was upregulated in the cortex, HC, and HT regions in C57Bl/6 mice infected with *P. berghei* ANKA [40]. Our findings are important because the IFN-*γ* receptor is widely expressed in cells of the central nervous system and provides many targets for this cytokine during CM, such as endothelial and astrocyte activation, parasitised red blood cell sequestration, blood brain barrier permeabilization, and CD8^+^ T cell accumulation [36, 41]. Interestingly, IFN-*γ* is a key immune mediator that controls the accumulation of *P. berghei*-infected red blood cells in brain tissues [42]. Infected red blood cells and malaria toxins induce immune cells to increase the synthesis of proinflammatory cytokines, such as TNF-*α* and IFN-*γ* [43]. These cytokines upregulate endothelial receptors, such as ICAM-1, which enhances the sequestration of immune cells in the brain. ICAM-1 also binds adherent parasitised red blood cells [44]; therefore, both the mechanical and immunological hypotheses work simultaneously.

The downregulation of IL-1*β* that we detected in the HC, FC, and LC of GX-infected female mice suggests that female gonadal steroids are involved in regulating inflammatory responses in these specific brain regions. Our results contrast with those of Miranda et al. [45] who found high proinflammatory cytokine expression in the HC and FC regions of *P. berghei* ANKA-infected intact female mice. These differences are most likely due to two factors. First, Miranda et al. used the C57Bl/6 mouse strain, and we used CBA/Ca mice. Second, Miranda's group collected brain samples on day 5 postinfection, while we performed the experiments 9 days after infection; the mRNA expression of cytokines could be altered after 4 days. These brain areas are involved in cognitive and behavioural alterations during the acute phase of experimental CM [46].

In our previous work, we measured IFN-*γ* and TNF-*α* mRNA expression in blood and quantified the concentration of both cytokines in the serum of intact, sham-, and GX-infected mice. Infected females exhibited higher levels of both cytokines than their male counterparts [17]. In the present work, we evaluated the mRNA expression of both IFN-*γ* and TNF-*α* in different brain areas because these cytokines are involved in both parasite elimination and development of cerebral malaria. In most of the brain areas assessed, the levels of IFN-*γ* and TNF-*α* were higher in intact infected males than in intact infected females. We also assessed the possibility that IL-10 downregulates the mRNA expression levels of both IFN-*γ* and TNF-*α* in POA, HT, HC, OB, FC, and LC; however, we were unable to detect IL-10 mRNA expression in the brain areas evaluated. These findings suggest that mechanisms that regulate the expression of both cytokines are different in blood and brain. It is possible that the increased IFN-*γ* and TNF-*α* mRNA expression and protein levels detected in blood and serum decreased parasitaemia on day 9 postinfection, and therefore, IFN-*γ* and TNF-*α* mRNA expression levels were downregulated in most of the brain areas assessed. These variations between sexes at least partially explain why infected males are more susceptible to developing cerebral malaria than infected female mice.

All these findings support the crucial importance of IFN-*γ* and TNF-*α* mRNA regulation in CM and the potential use of gonadal steroids or their derivatives in CM immunomodulation.

On the other hand, nitric oxide is a vasodilator that inhibits platelet aggregation [47]. In CM, the suppression of nitric oxide synthesis increased with disease severity [27, 48]. We found that *P. berghei* ANKA-infected female mice exhibited higher serum levels of nitric oxide than both intact and GX males, which may at least partially explain the lower severity of *P. berghei* ANKA infection in females. Our findings are consistent with those of Mwanga-Amumpaire et al. and Hawkes et al. who used nitric oxide inhalation as an adjunctive therapy for severe malaria because it decreases endothelial inflammation and reduces the adhesion of parasitised erythrocytes [48–50].

## 5. Conclusions

Gonadal hormones, cytokines, endothelial cells and nitric oxide act together in both systemic immune responses and in the brain in a multifaceted manner, influencing neuroinflammation [51]. In our model of cerebral malaria, cytokine mRNA expression patterns in the brain were influenced by sex; thus, therapeutic manipulation of the immune system may affect brain function. Further work is needed to determine the exact functions of cytokines in each brain region and whether other factors are associated with parasites or hosts in the CM model used herein.

## Data Availability

The data used to support the findings of this study are available from the corresponding author upon request.
